# The Danish Nationwide Electrocardiogram (ECG) Cohort

**DOI:** 10.1007/s10654-024-01105-9

**Published:** 2024-02-26

**Authors:** Christoffer Polcwiartek, Mikkel Porsborg Andersen, Helle Collatz Christensen, Christian Torp-Pedersen, Kathrine Kold Sørensen, Kristian Kragholm, Claus Graff

**Affiliations:** 1 Department of Cardiology, Aalborg University Hospital, Hobrovej 18-22, Aalborg, DK-9000 Denmark; 2 Department of Cardiology, Nordsjællands Hospital, Hillerød, Denmark; 3 Prehospital Center, Region Zealand, Næstved, Denmark; 4 Department of Clinical Medicine, University of Copenhagen, Copenhagen, Denmark; 5 Department of Public Health, University of Copenhagen, Copenhagen, Denmark; 6 Unit of Clinical Biostatistics and Epidemiology, Aalborg University Hospital, Aalborg, Denmark; 7 Department of Health Science and Technology, Aalborg University, Aalborg, Denmark

**Keywords:** Danish nationwide administrative registers, Electrocardiogram, ECG, In-hospital, Population-based, Pre-hospital

## Abstract

The electrocardiogram (ECG) is a non-invasive diagnostic tool holding significant clinical importance in the diagnosis and risk stratification of cardiac disease. However, access to large-scale, population-based digital ECG data for research purposes remains limited and challenging. Consequently, we established the Danish Nationwide ECG Cohort to provide data from standard 12-lead digital ECGs in both pre- and in-hospital settings, which can be linked to comprehensive Danish nationwide administrative registers on health and social data with long-term follow-up. The Danish Nationwide ECG Cohort is an open real-world cohort including all patients with at least one digital pre- or in-hospital ECG in Denmark from January 01, 2000, to December 31, 2021. The cohort includes data on standardized and uniform ECG diagnostic statements and ECG measurements including global parameters as well as lead-specific measures of waveform amplitudes, durations, and intervals. Currently, the cohort comprises 2,485,987 unique patients with a median age at the first ECG of 57 years (25th–75th percentiles, 40–71 years; males, 48%), resulting in a total of 11,952,430 ECGs. In conclusion, the Danish Nationwide ECG Cohort represents a novel and extensive population-based digital ECG dataset for cardiovascular research, encompassing both pre- and in-hospital settings. The cohort contains ECG diagnostic statements and ECG measurements that can be linked to various nationwide health and social registers without loss to follow-up.

## Introduction

The electrocardiogram (ECG) remains one of the most important clinical tools used for the diagnostic evaluation of cardiac disease. The ECG is widely available, non-invasive, and inexpensive, and standard ECG testing is performed with 12 leads and records cardiac electrical activity usually over 10 s [1]. An abnormal ECG is often a marker of direct cardiac pathology and may be suggestive of myocardial infarction or ischemia, left ventricular hypertrophy, bundle branch block, or tachy- and bradyarrhythmias [2–4].

The ECG holds valuable clinical information that may be utilized in cardiovascular risk assessment and prognostication both in the clinical setting as well as for research purposes [5]. For instance, the ECG can be used to identify novel ECG markers indicative of early-stage cardiac disease or to explore the efficacy of machine learning models that may have the potential to promote cardiovascular health and prevent future cardiovascular disease events [6]. A major limitation and challenge in this setting is the lack of large-scale uniform digital ECG data from e.g., nationwide cohorts that can be linked to concurrent health and social data with long-term follow-up of patients.

We have filled this gap by establishing the Danish Nationwide ECG Cohort. This paper provides a demographical description of the cohort, gives insight into strengths and limitations of the cohort, and describes how the cohort can be linked to other Danish nationwide administrative registers on blood test results, comorbidities, medication use, procedures, socioeconomic status, and long-term clinical outcomes including mortality data.

## Methods

### The Danish research setting

Denmark covers approximately 42.952 km^2^ divided into 5 regions (i.e., the North Denmark Region, Central Denmark Region, Region of Southern Denmark, Region Zealand, and Capital Region of Denmark) and 98 municipalities [7, 8]. The annual Danish residential population was approximately 5.9 million individuals in 2023 [9]. The Danish healthcare system is free of charge as it is funded by the Danish tax system and managed by the central government, regions, and municipalities.

Denmark has a long history of recording health and social data on Danish residents, gathering this into various nationwide administrative registers. The Danish Civil Registration System, established in 1968, introduced the unique civil personal registration (CPR) number to all Danish residents, which has since been given to Danish residents either at birth or upon immigration [10]. The CPR number is included in all Danish nationwide administrative registers, making it possible to link information across different registers to the individual as well as other data sources if the CPR number is available. Most of the common Danish nationwide administrative registers have been previously described in detail elsewhere [10–18].

In accordance with the Danish legislation, it is possible to use the registers if the purpose is for scientific and statistical research of significant societal importance [19]. The variety of health, social, and economic data with long-term follow-up available from the registers provide a unique possibility to perform nationwide register-based studies on the Danish population.

### Information in the Danish Nationwide ECG Cohort

The Danish Nationwide ECG Cohort is an open real-world cohort containing information on high-quality standard 12-lead digital ECG recordings from pre- and in-hospital settings in Denmark, in which prehospital refers to ambulance ECGs and in-hospital to in- and outpatient ECGs. The cohort includes both pediatric and adult patients with at least one ECG examination performed between January 01, 2000, and December 31, 2021, and is planned to undergo continuous updates with new ECGs from all Danish regions that is scheduled to occur annually. Of note, ECG data from primary care and private hospitals or clinics are not available in this cohort as these services are privatized and not operated by the Danish Regions.

All ECG data are stored securely on servers at Statistics Denmark, and data access is only accessible through affiliation to a Danish research institution, ensuring compliance with data protection regulations and preventing public access [20]. Thus, datasets cannot be made publicly available. Researchers that wish to access the data and programming codes can contact the authors of this study for collaboration on further studies.

The Danish Nationwide ECG Cohort contains information about the CPR number of patients, the date and time of ECG acquisition, the setting where the ECG was acquired, and in which region the ECG examination was performed. Furthermore, standardized ECG diagnostic statements and ECG measurements encompassing both global parameters and lead-specific measures of waveform amplitudes, durations, and intervals are available.

In the present paper, the Danish Civil Registration System has been used to report demographic data for the cohort description [10], and patients were not included in case of missing data on age or sex. Furthermore, data on vital status were obtained from the Danish Cause of Death Register to ensure that the included patients were not erroneously recorded with a death date prior to the ECG recording date [12].

As stated previously, it is possible to link the ECG data to all of the other Danish nationwide administrative registers through the CPR number to obtain health and social data such as blood test results, comorbidities, medication use, procedures, income, education, employment, and long-term clinical outcomes including mortality data.

Data analysis was performed using R version 4.2.1 (R Core Team, Vienna, Austria).

### Acquisition, processing, and analysis of ECG data

In both the pre- and in-hospital setting, trained healthcare personnel followed a standardized protocol to digitally record standard 12-lead ECGs, preferably while the patient was at rest and in supine position. All ECG data were stored in the MUSE Cardiology Information System (GE Healthcare, Wauwatosa, WI, USA). To ensure consistency and avoid discrepancies caused by different algorithms and vendors during the ECG data sampling period, the Marquette 12SL algorithm version 23 was utilized to reanalyze all ECG data [21]. This process ensured standardized and uniform ECG diagnostic statements as well as global and lead-specific ECG measurements.

All ECGs were filtered to within the band between 0.16 and 150 Hz. The Marquette 12SL Hookup Advisor was utilized to evaluate the quality of ECG leads and assign them to three levels (i.e., green, yellow, or red) based on factors such as muscle tremor, baseline sway, AC interference, electrode noise, and lead saturation [21]. We excluded ECGs flagged as red from further analysis, as were ECGs that were incompatible with the Marquette 12SL algorithm reanalysis, displayed flatline recordings in any lead, had a heart rate of 0 beats per minute, deviated from the standard 12-lead configuration, were shorter than 10 s, had a sampling rate below 500 Hz, were duplicates, or had invalid CPR numbers preventing linkage to Danish nationwide administrative registers. For the remaining ECGs, the MUSE Cardiology Information System, along with the Marquette 12SL algorithm, generated standardized ECG diagnostic statements enabling the identification of commonly encountered ECG abnormalities. These statements align with the recommendations of the AHA/ACC/HRS (i.e., the American Heart Association, the American College of Cardiology, and the Heart Rhythm Society) [22].

**Fig. 1:**
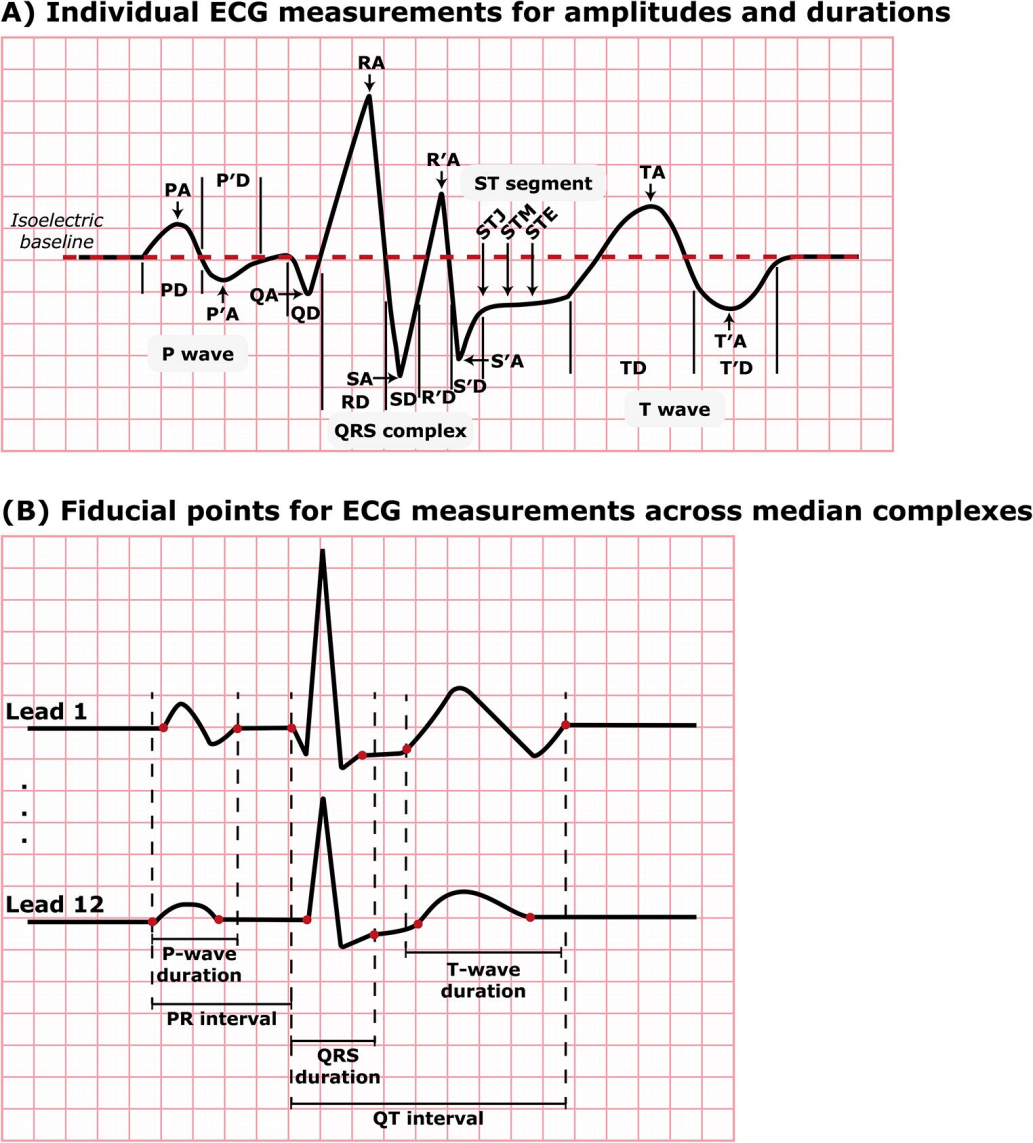
The ECG reflects specific cardiac events and electrical activities. The P wave represents atrial depolarization, initiated by electrical impulses from the sinoatrial node. The PR interval represents conduction through the atrioventricular node. The QRS complex represents ventricular depolarization, marking the onset of systole and ventricular contraction. The T wave represents ventricular repolarization, with the ST segment representing an electrically neutral phase between ventricular depolarization (QRS complex) and repolarization (T wave). Finally, the QT interval represents the time taken for both ventricular depolarization and repolarization, effectively marking the time from ventricular isovolumetric contraction to relaxation. (**A**) The P and T waves can exhibit either unipolar or bipolar morphologies, resulting in positive, negative, or zero values for P, P′, T, and T′ waves depending on the waveform configuration. Due to the standard definition of the Q, S, and S′ waves as negative deflections, their amplitudes are represented as positive values, with the implicit understanding that they are negative deflections. STJ, commonly referred to as the J point, is defined as the ST level at QRS offset relative to QRS onset. The ST level at the QRS offset plus 1/16 of the average RR interval represents STM. Similarly, STE refers to the ST level at the QRS offset plus 1/8 of the average RR interval. (**B**) As opposed to human readers who may only inspect the QRS duration in any single lead of the ECG, the Marquette 12SL algorithm measures global intervals from the earliest to the latest waveform deflection across all 12 leads as represented by the red dots

To obtain ECG measurements encompassing both global parameters, such as heart rate, P-wave duration, PR interval, QRS duration, QT interval, corrected QT interval, frontal axis of P, QRS, and T waves, as well as lead-specific measures of waveform amplitudes, durations, and intervals, the Marquette 12SL algorithm generates a representative median beat in each lead, formed by aligning similar-shaped P-QRS-T complexes. Subsequently, fiducial points, including onset, offset, and peak points of ECG waveforms and segments, are derived from the temporally aligned complexes and utilized for the calculation of waveform amplitudes and durations. The Marquette 12SL algorithm adjusts the median complex in such a way that the voltage at the QRS onset is defined as 0. Consequently, all lead-specific amplitudes and ST levels are measured in µV relative to the voltage at the QRS onset (Fig. 1A). In addition, the global intervals measured by the 12SL algorithm represent the duration between the earliest and latest waveform deflection observed in any lead (Fig. 1B). Waveform areas computed by the Marquette 12SL algorithm require multiplication by 19.52 µV⋅ms to make the areas comparable to area measurements by non-GE Healthcare software [23, 24]. A comprehensive description of all derived ECG variables in the Danish Nationwide ECG Cohort is reported in Table 1.

**Table 1:** List of variables included in the Danish Nationwide ECG Cohort

Variable name	Description	Unit
PatientID	Pseudonymized CPR number	
Location	Region codes to identify in which Danish region/hospital department the ECG was acquired	
Acq_Date	Date of ECG recording	
Acq_Time	Time of ECG recording	
AnalysisSoftwareVersion	Marquette 12SL algorithm version 23	
ECGSampleBase	ECG sampling rate	
NumQRSComplexes	Total number of QRS complexes in the ECG	
Statements	Diagnostic statements generated by the Marquette 12SL algorithm version 23	
**Global ECG measurements**		
AvgRRInterval	Average R-R interval	ms
VentricularRate	Heart rate	bpm
AtrialRate	Atrial heart rate	bpm
PR_Interval	PR interval	ms
QRSDuration	QRS duration	ms
QT_Interval	QT interval	ms
QTcBazett	QT interval corrected based on the Bazett formula	ms
QTcFridericia	QT interval corrected based on the Fridericia formula	ms
QTcFramingham	QT interval corrected based on the Framingham formula	ms
P_Frontal_Axis	P-wave axis frontal plane	degrees
R_Frontal_Axis	R-wave axis frontal plane	degrees
T_Frontal_Axis	T-wave axis frontal plane	degrees
QOnset	Q-wave onset	sample #
QOffset	Q-wave offset	sample #
POnset	P-wave onset	sample #
POffset	P-wave offset	sample #
TOffset	T-wave onset	sample #
**Lead-specific ECG measurements**		
P_OnsetAmpl_X	P-wave onset amplitude in lead X	µV
P_PeakAmpl_X	P-wave peak amplitude in lead X	µV
P_Duration_X	P-wave duration in lead X	ms
P_Area_X	P-wave area in lead X	µV⋅ms
P_PeakTime_X	P-wave peak time (from POnset) in lead X	ms
PP_PeakAmpl_X	P′-wave peak amplitude in lead X	µV
PP_Duration_X	P′-wave duration in lead X	ms
PP_Area_X	P′-wave area in lead X	µV⋅ms
PP_PeakTime_X	P′-wave peak time (from P′Onset) in lead X	ms
Q_PeakAmpl_X	Q-wave peak amplitude in lead X	µV
Q_Duration_X	Q-wave duration in lead X	ms
Q_Area_X	Q-wave area in lead X	µV⋅ms
Q_PeakTime_X	Q-wave peak time (from QOnset) in lead X	ms
R_PeakAmpl_X	R-wave peak amplitude in lead X	µV
R_Duration_X	R-wave duration in lead X	ms
R_Area_X	R-wave area in lead X	µV⋅ms
R_PeakTime_X	R-wave peak time (from ROnset) in lead X	ms
S_PeakAmpl_X	S-wave peak amplitude in lead X	µV
S_Duration_X	S-wave duration in lead X	ms
S_Area_X	S-wave area in lead X	µV⋅ms
S_PeakTime_X	S-wave peak time (from SOnset) in lead X	ms
RP_PeakAmpl_X	R′-wave peak amplitude in lead X	µV
RP_Duration_X	R′-wave duration in lead X	ms
RP_Area_X	R′-wave area in lead X	µV⋅ms
RP_PeakTime_X	R′-wave peak time (from R′Onset) in lead X	ms
SP_PeakAmpl_X	S′-wave peak amplitude in lead X	µV
SP_Duration_X	S′-wave duration in lead X	ms
SP_Area_X	S′-wave area in lead X	µV⋅ms
SP_PeakTime_X	S′-wave peak time (from S′Onset) in lead X	ms
STJ_X	ST-segment level at the J point of the terminal QRS complex in lead X	µV
STM_X	ST-segment level at the QRS offset plus 1/16 of the average RR interval in lead X	µV
STE_X	ST-segment level at the QRS offset plus 1/8 of the average RR interval in lead X	µV
MaxST_X	Maximum of STJ_X, STM_X, and STE_X in lead X	µV
MinST_X	Minimum of STJ_X, STM_X, and STE_X in lead X	µV
T_Special_X	Minimum of either T-wave amplitude or (T-wave amplitude - STE_X) in lead X	µV
QRS_Balance_X	Maximum R-wave amplitude - maximum S-wave amplitude in lead X	µV
QRS_Deflection_X	QRS peak-to-peak amplitude in lead X (R_Amp_X - S_Amp_X)	µV
Max_R_Ampl_X	Maximum of the R-wave or R′-wave in lead X	µV
Max_S_Ampl_X	Maximum of the Q-wave, S-wave, or S′-wave in lead X	µV
T_PeakAmpl_X	T-wave peak amplitude in lead X	µV
T_Duration_X	T-wave duration in lead X	ms
T_Area_X	T-wave area in lead X	µV⋅ms
T_PeakTime_X	T-wave peak time (from TOnset) in lead X	ms
TP_PeakAmpl_X	T′-wave peak amplitude in lead X	µV
TP_Duration_X	T′-wave duration in lead X	ms
TP_Area_X	T′-wave area in lead X	µV⋅ms
TP_PeakTime_X	T′-wave peak time (from T′Onset) in lead X	ms
T_EndAmpl_X	T-wave offset amplitude in lead X	µV
PFull_Area_X	Sum of P-wave and P′-wave areas in lead X	µV⋅ms
QRS_Area_X	QRS area in lead X	µV⋅ms
TFull_Area_X	Sum of T-wave and T′-wave areas in lead X	µV⋅ms

bpm, beats per minute; CPR, civil personal registration; ECG, electrocardiogram

The digital waveforms are also available as Extensible Markup Language (.XML) files containing the waveform data as well as global and lead-specific measurements. GE Healthcare supported the export of ECGs to .XML files during the data collection phase, ensuring that all ECGs could undergo reanalysis using the same version of the Marquette 12SL algorithm. GE Healthcare also assisted in exporting the reanalyzed ECGs to make the .XML files processed by the same version of 12SL accessible.

Several studies have leveraged the stability and accuracy of the Marquette 12SL algorithm for ECG measurements including amplitudes, durations, and intervals [25], and there is little evidence to suggest that manual methods are advantageous for large clinical trials or epidemiological studies compared with automated methods.

Details and criteria on the ECG diagnostic statements and ECG measurements, as generated by the Marquette 12SL algorithm, have been published in detail previously [21, 25].

### Ethics approval

Approval to collect the nationwide ECG data was granted by the Record Data Team, Center for Health in the Capital Region of Denmark (approval number: R-21032357). Additional approval to process the data sources for statistics and scientific research purposes was granted by the data responsible institute of the Capital Region of Denmark (approval number: P-2019-533) in compliance with both the Danish Data Protection Act and the General Data Protection Regulation [19, 26].

## Results

**Fig. 2:**
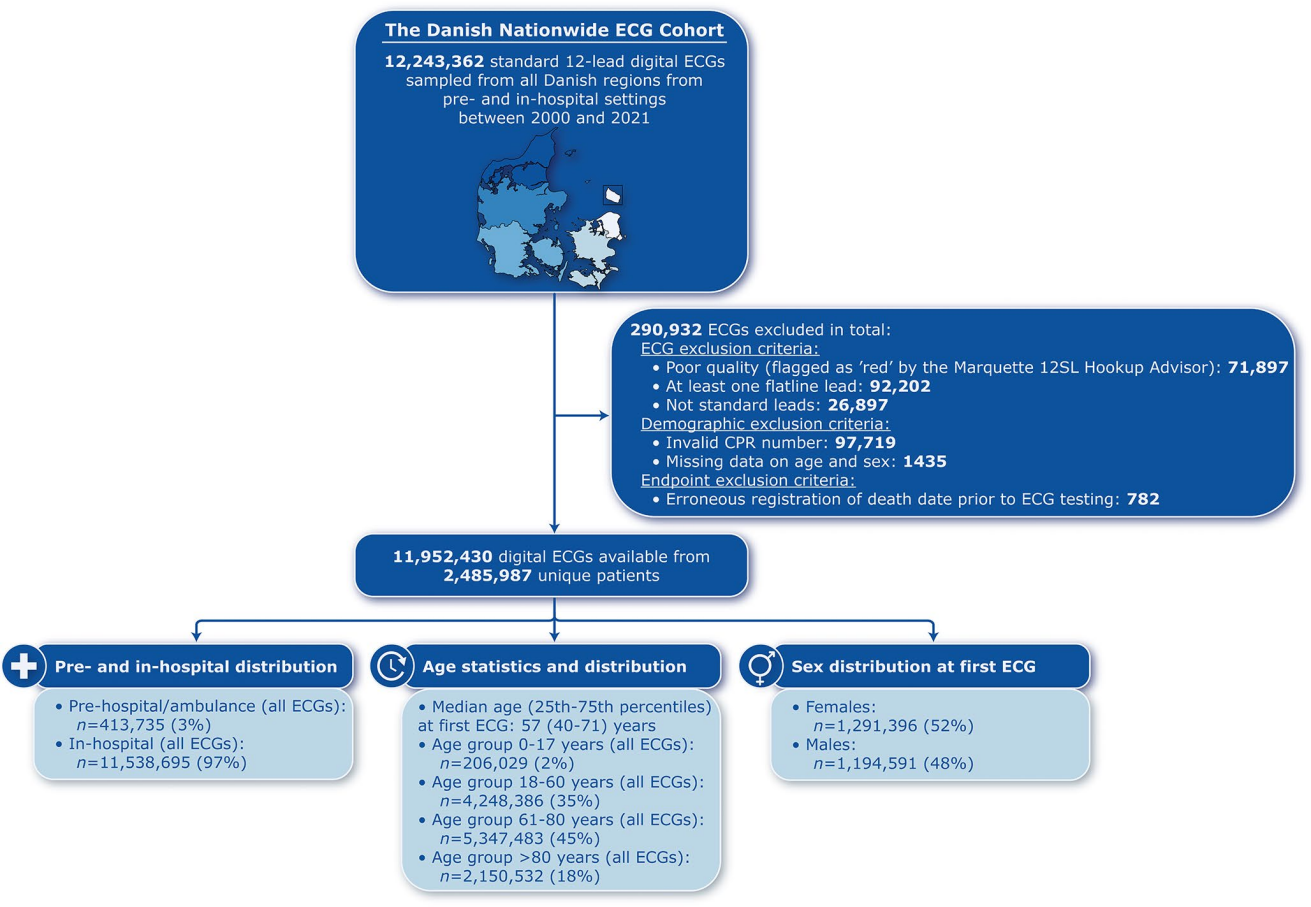
Flowchart of the patient selection and ECG data sampling process with highlighted demographic characteristics of the cohort

The Danish Nationwide ECG Cohort encompassed 11,952,430 ECG recordings derived from 2,485,987 unique patients from pre- and in-hospital settings. Figure 2 illustrates the patient selection and ECG data sampling process. Among the total ECGs, 413,735 (3%) were acquired in prehospital settings, while 11,538,695 (97%) originated from in-hospital settings. The median age of patients at initial ECG testing was 57 (25th–75th percentiles, 40–71) years. Approximately 2% of all ECGs were sampled from the pediatric population (0–17 years), while the predominant age group for ECG recordings was 61–80 years (45%). The distribution of male and female patients in the cohort was generally balanced, with females comprising 52% and males 48%.

**Fig. 3:**
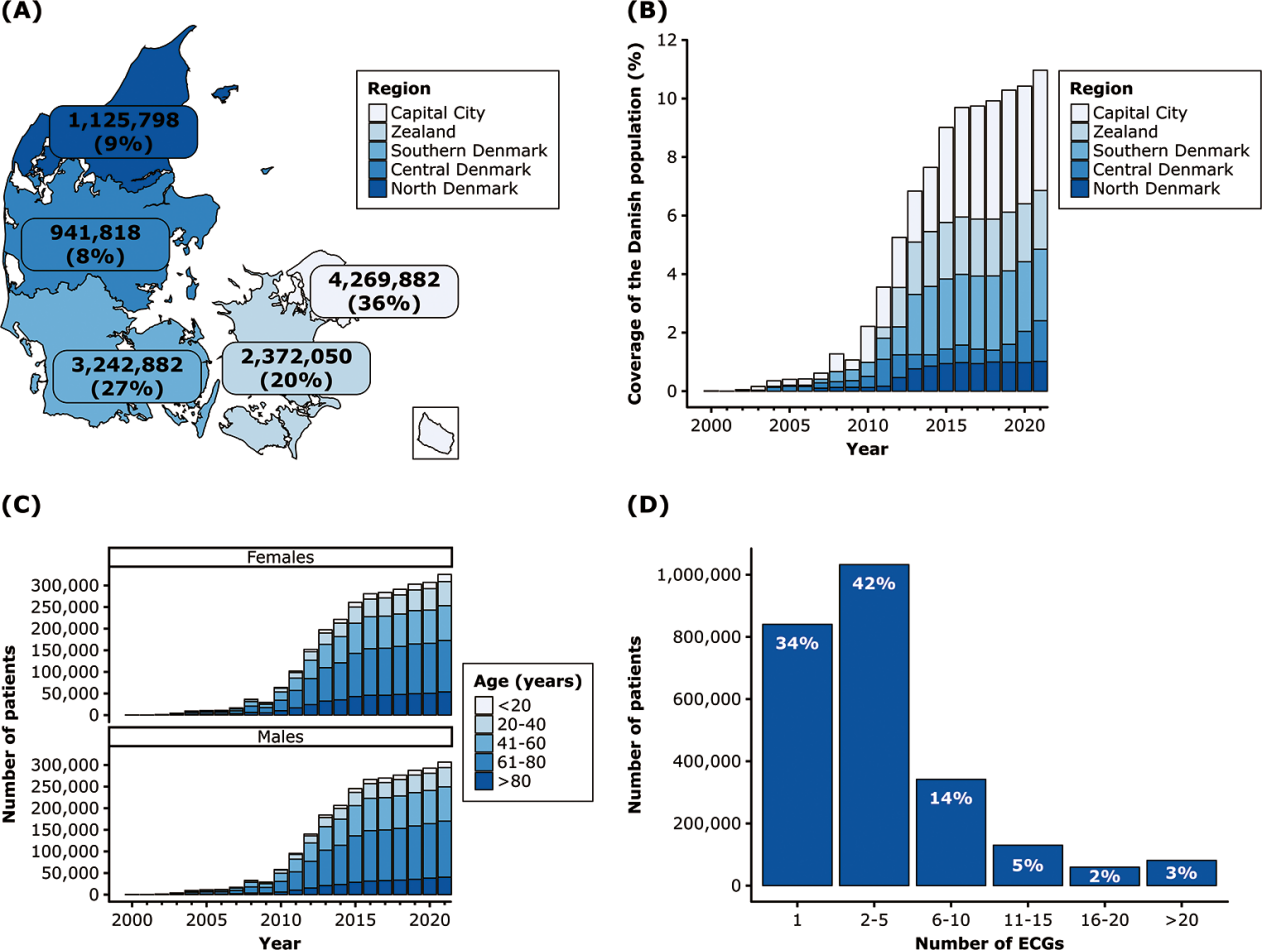
(**A**) Denmark map with the total number and percentage of ECGs for each region. (**B**) Barplot of the ECG data coverage by region according to the Danish population for each year of the study period. (**C**) The number of patients with ECG data stratified by age and sex. (**D**) The number of ECGs per patient in the cohort

The total number of ECGs stratified by all regions is displayed in Fig. 3A. The majority of ECGs were acquired from the Capital Region of Denmark (36%), with fewest ECG recordings being obtained in the Central Denmark Region (8%). The distribution of ECGs across all regions for each sampling year is displayed in Fig. 3B. Over time, the ECG data coverage (i.e., ratio of unique patients in a year to the Danish population that year) increased from < 1% in 2000 to 11% in 2021. As before, most ECGs were acquired from the Capital Region of Denmark. The age and sex distribution of the ECG data is displayed in Fig. 3C. Overall, no clinically meaningful differences were observed in the number of ECGs between female and male patients. The majority of ECG examinations were performed in patients aged 40–80 years. Finally, Fig. 3D depicts the total number of ECGs per patient and highlights that it is possible to study temporal ECG changes as 42% of the patients in the cohort have undergone between 2 and 5 ECG examinations during the study period.

## Discussion

The Danish Nationwide ECG Cohort is, to our knowledge, the largest population-based, real-world digital ECG dataset globally that can be linked to comprehensive nationwide register-based data such as blood test results, comorbidities, medication use, procedures, income, education, employment, and long-term clinical outcomes including mortality data. The cohort provides important research possibilities for improving and optimizing cardiovascular risk stratification and prognostication as well as identifying novel ECG markers. Interestingly, the cohort holds an exciting potential for machine learning models trained on a widely used diagnostic tool like the ECG. This can be integrated with regularly collected register-based data to transform high-throughput diagnostics across various cardiovascular diseases.

Several large 12-lead ECG datasets have previously been made publicly available, significantly advancing the real-world assessment of ECG markers and their prognostic relevance in cardiovascular disease [27]. Databases such as the CHARGE Consortium [28] and the UK Biobank [29] provide detailed phenotyping of hundreds of thousands of participants followed over time, generating data on lifestyle, environmental, and genetic factors to explore their roles in cardiovascular disease development and progression. The detailed phenotyping within these databases offers a comprehensive understanding of non-ECG characteristics and cardiovascular outcomes. In contrast, our ECG cohort, encompassing the entire Danish population in both pre- and in-hospital settings, stands out with its population-based unselected approach. This not only enhances the generalizability of findings to the broader population but also provides insights that may be relevant in non-clinical and non-acute settings. Furthermore, the comprehensive 20-year study period in our cohort offers a unique temporal aspect, enabling investigation of long-term cardiovascular trends and outcomes.

Although routine ECG screening of asymptomatic adults at low risk of cardiovascular disease events is not recommended [30], the various indications for ECG testing in pre- and in-hospital settings were not present in this cohort, which is an important limitation. In addition, ECG abnormalities that may be used for prediction of long-term clinical outcomes in epidemiological studies may not necessarily be directly related to underlying cardiac pathology and can be attributed to comorbidities, electrolyte disturbances, medication use, and lifestyle factors of patients that are naturally correlated with cardiovascular disease. Although most patients undergo ECG testing at some point during their lifetime, there is still a potential for selection bias in our cohort as it is restricted to patients who had at least one pre- or in-hospital ECG during the comprehensive 20-year study period, and we lack data on the source population from which patients with ECGs were sampled. This is important as certain demographic and clinical characteristics may be over- or underrepresented in the cohort. It is also important to note that certain limitations may arise from when ECG data are linked with health and social data in case of missing covariates, and that data on an individual level such as specific ECGs cannot be accessed through Statistics Denmark. Altogether, researchers should therefore address these limitations carefully when working with the cohort.

The main strength of the Danish Nationwide ECG Cohort is that all ECGs are collected from pre- and in-hospital settings across Denmark, thereby representing a large variety of patients during an extensive study period. This may minimize the risk of selection bias and makes findings from this cohort representative. Furthermore, the ECG data are collected as part of the Danish public healthcare system that is free and accessible for all. The information is reported uniformly through electronic reporting systems, ensuring data consistency and enabling linkage with other important health and social data. Another strength of this cohort is that all ECG data have been reanalyzed using the Marquette 12SL algorithm version 23 to obtain standardized and uniform diagnostic statements and measurements for all ECGs throughout the study period. Diagnostic statements and fiducial points on the ECG generated by the Marquette 12SL algorithm have a high accuracy and validity [25]. For example, the diagnostic statement ‘complete left bundle branch block’ has been favorably validated, with a specificity from 99.9 to 100% and sensitivity from 78 to 90.9% when compared to a diagnosis by cardiologists using traditional criteria [31]. However, all diagnostic statements in this cohort have not been overread by cardiologists and thereby potentially modified. In the clinical setting, ECGs with only minor abnormalities may have prompted immediate cardiology assessment and evaluation and vice versa for major ECG abnormalities. Furthermore, it is noteworthy that the cohort can be linked with complete long-term follow-up data on clinical outcomes, and for the majority of patients, we have unique opportunities to follow serial ECG changes over time and relate these to cardiovascular disease outcomes and prognosis.

## Conclusions

In conclusion, the Danish Nationwide ECG Cohort represents a novel and extensive population-based digital ECG dataset for cardiovascular research, encompassing both pre- and in-hospital settings. The cohort contains ECG diagnostic statements and ECG measurements that can be linked to various nationwide health and social registers without loss to follow-up.

## References

[1] Kligfield P, Gettes LS, Bailey JJ (2007). Recommendations for the standardization and interpretation of the electrocardiogram: part I: the electrocardiogram and its technology: a scientific statement from the American Heart Association Electrocardiography and Arrhythmias Committee, Council on Clinical Cardiology; the American College of Cardiology Foundation; and the Heart Rhythm Society: endorsed by the International Society for Computerized Electrocardiology. Circulation.

[2] Hancock EW, Deal BJ, Mirvis DM (2009). AHA/ACCF/HRS recommendations for the standardization and interpretation of the electrocardiogram: part V: electrocardiogram changes associated with cardiac chamber hypertrophy: a scientific statement from the American Heart Association Electrocardiography and Arrhythmias Committee, Council on Clinical Cardiology; the American College of Cardiology Foundation; and the Heart Rhythm Society: endorsed by the International Society for Computerized Electrocardiology. Circulation.

[3] Surawicz B, Childers R, Deal BJ (2009). AHA/ACCF/HRS recommendations for the standardization and interpretation of the electrocardiogram: part III: intraventricular conduction disturbances: a scientific statement from the American Heart Association Electrocardiography and Arrhythmias Committee, Council on Clinical Cardiology; the American College of Cardiology Foundation; and the Heart Rhythm Society: endorsed by the International Society for Computerized Electrocardiology. Circulation.

[4] Thygesen K, Alpert JS, Jaffe AS (2018). Fourth Universal Definition of Myocardial Infarction (2018). Circulation.

[5] Jørgensen PG, Jensen JS, Marott JL, Jensen GB, Appleyard M, Mogelvang R (2014). Electrocardiographic changes improve risk prediction in asymptomatic persons age 65 years or above without cardiovascular disease. J Am Coll Cardiol.

[6] Somani S, Russak AJ, Richter F (2021). Deep learning and the electrocardiogram: review of the current state-of-the-art. Europace.

[7] Statistics Denmark, Area. 2024. https://www.dst.dk/en/Statistik/emner/miljoe-og-energi/areal. Accessed 01/18/2024.

[8] Statistics Denmark. Regions, provinces and municipalities. 2024. https://www.dst.dk/en/Statistik/dokumentation/nomenklaturer/nuts. Accessed 01/18/2024.

[9] Statistics Denmark. Population figures. 2024. https://www.dst.dk/en/Statistik/emner/borgere/befolkning/befolkningstal. Accessed 01/18/2024.

[10] Pedersen CB (2011). The Danish Civil Registration System. Scand J Public Health.

[11] Baadsgaard M, Quitzau J (2011). Danish registers on personal income and transfer payments. Scand J Public Health.

[12] Helweg-Larsen K (2011). The Danish Register of causes of Death. Scand J Public Health.

[13] Kildemoes HW, Sorensen HT, Hallas J (2011). The Danish national prescription Registry. Scand J Public Health.

[14] Lynge E, Sandegaard JL, Rebolj M (2011). The Danish National Patient Register. Scand J Public Health.

[15] Mors O, Perto GP, Mortensen PB (2011). The Danish Psychiatric Central Research Register. Scand J Public Health.

[16] Petersson F, Baadsgaard M, Thygesen LC (2011). Danish registers on personal labour market affiliation. Scand J Public Health.

[17] Arendt JFH, Hansen AT, Ladefoged SA, Sorensen HT, Pedersen L, Adelborg K (2020). Existing data sources in clinical epidemiology: Laboratory Information System databases in Denmark. Clin Epidemiol.

[18] Schmidt M, Schmidt SAJ, Adelborg K (2019). The Danish health care system and epidemiological research: from health care contacts to database records. Clin Epidemiol.

[19] Justitsministeriet. Lov Om supplerende bestemmelser til forordning om beskyttelse af fysiske personer i forbindelse med behandling af personoplysninger og om fri udveksling af sådanne oplysninger (databeskyttelsesloven). 2018. https://www.retsinformation.dk/eli/lta/2018/502. Accessed 01/18/2024.

[20] Statistics Denmark. Access to data. 2024. https://www.dst.dk/en/TilSalg/Forskningsservice/Dataadgang. Accessed 01/18/2024.

[21] Marquette™ 12SL™ ECG Analysis Program - Physician’s Guide. 2022. https://www.gehealthcare.com/support/manuals?search=eyJzZWFyY2hUZXJtIjoiMjA1NjI0Ni0wMDciLCJsYW5ndWFnZU5hbWUiOiJFbmdsaXNoIChFTikifQ%3D%3D. Accessed 01/18/2024.

[22] Mason JW, Hancock EW, Gettes LS (2007). Recommendations for the standardization and interpretation of the electrocardiogram: part II: Electrocardiography diagnostic statement list: a scientific statement from the American Heart Association Electrocardiography and Arrhythmias Committee, Council on Clinical Cardiology; the American College of Cardiology Foundation; and the Heart Rhythm Society: endorsed by the International Society for Computerized Electrocardiology. Circulation.

[23] Snyder ML, Soliman EZ, Whitsel EA, Gellert KS, Heiss G (2014). Short-term repeatability of electrocardiographic P wave indices and PR interval. J Electrocardiol.

[24] Soliman EZ. Response to Letter by Dewhurst and Adams. Stroke. 2011;42(2).

[25] Marquette™ 12SL™ ECG Analysis Program - Statement of Validation and Accuracy. 2008. https://www.gehealthcare.com/support/manuals?search=eyJzZWFyY2hUZXJtIjoiNDE2NzkxLTAwMyIsImxhbmd1YWdlTmFtZSI6IkVuZ2xpc2ggKEVOKSJ9. Accessed 01/18/2024.

[26] Radley-Gardner O, Beale H, Zimmermann R. Fundamental texts on European private law. Hart Publishing; 2016.

[27] Liu H, Chen D, Chen D (2022). A large-scale multi-label 12-lead electrocardiogram database with standardized diagnostic statements. Sci Data.

[28] Psaty BM, O’Donnell CJ, Gudnason V (2009). Cohorts for heart and Aging Research in genomic epidemiology (CHARGE) Consortium: design of prospective meta-analyses of genome-wide association studies from 5 cohorts. Circ Cardiovasc Genet.

[29] Sudlow C, Gallacher J, Allen N (2015). UK biobank: an open access resource for identifying the causes of a wide range of complex diseases of middle and old age. PLoS Med.

[30] Curry SJ, Krist AH, Owens DK (2018). Screening for cardiovascular disease risk with electrocardiography: US Preventive Services Task Force Recommendation Statement. JAMA.

[31] Andersen DC, Kragholm K, Petersen LT (2021). Association between vectorcardiographic QRS area and incident heart failure diagnosis and mortality among patients with left bundle branch block: a register-based cohort study. J Electrocardiol.

